# TELEMEDICINE CONSULTATIONS IN SWEDEN 2020–2022: EXPLORING AGE DIFFERENCES

**DOI:** 10.1093/geroni/igad104.2315

**Published:** 2023-12-21

**Authors:** Felicia Gabrielsson-Järhult, Ingemar Kåreholt

**Affiliations:** Jönköping University, Jönköping, Jonkopings Lan, Sweden; Jönköping University, School of Health and Welfare, Jönköping, Jonkopings Lan, Sweden

## Abstract

Health care in Sweden is heavily subsidized and costs for the user are small, both for physical consultation and telemedicine, and for public and private health care. It has been argued that telemedicine consultations will increase care consumption, lead to higher costs, and create inequalities. As older people often are less skilled in digital technology, increased digitalization of care might increase inequalities. Telehealth is mainly conducted by private companies. This study is based on all private telemedicine consultations in two regions in southern Sweden 2020-2022, with approx. 656,000 inhabitants. 106,000 persons had 343,000 telemedicine consultations, 3.2 consultations p/p. The number of consultations differed neglectable between age groups, for women median was 2.0 for the age groups (< 65/65-79/80+), and for men it was 1.0. The proportion of people WITH telemedicine consultations differed heavily between the age group as well as months. Total number of telemedicine consultations per month varied 4-8.5 thousand among women, and 2-4 thousand among men. The months with most consultations were March-June 2021. Proportion of telehealth consultations was low in the age group 65-79, and even lower among 80+. The proportion of consultations by people 65-79 years increased steadily and reached its peak July-September 2021 with >20%. After that, it increased rapidly down to < 3% among women and < 4% among men. The pattern was similar among people 80+ with peaks at approx. 2% among women and close to 2% among men. The conclusion is that there is still a huge age difference in the use of telehealth consultations.

